# Identification of the *para*-nitrophenol catabolic pathway, and characterization of three enzymes involved in the hydroquinone pathway, in *pseudomonas *sp. 1-7

**DOI:** 10.1186/1471-2180-12-27

**Published:** 2012-03-02

**Authors:** Shuangyu Zhang, Wen Sun, Li Xu, Xiaomei Zheng, Xiaoyu Chu, Jian Tian, Ningfeng Wu, Yunliu Fan

**Affiliations:** 1 Biotechnology Research Institute, Chinese Academy of Agricultural Sciences, 12 Zhongguancun South Street, Haidian District, Beijing 100081, PR China

**Keywords:** *para*-Nitrophenol, Catabolism, Hydroquinone pathway, Hydroxyquinol pathway, *Pseudomonas*

## Abstract

**Background:**

*para*-Nitrophenol (PNP), a priority environmental pollutant, is hazardous to humans and animals. However, the information relating to the PNP degradation pathways and their enzymes remain limited.

**Results:**

*Pseudomonas *sp.1-7 was isolated from methyl parathion (MP)-polluted activated sludge and was shown to degrade PNP. Two different intermediates, hydroquinone (HQ) and 4-nitrocatechol (4-NC) were detected in the catabolism of PNP. This indicated that *Pseudomonas *sp.1-7 degraded PNP by two different pathways, namely the HQ pathway, and the hydroxyquinol (BT) pathway (also referred to as the 4-NC pathway). A gene cluster (*pdcEDGFCBA*) was identified in a 10.6 kb DNA fragment of a fosmid library, which cluster encoded the following enzymes involved in PNP degradation: PNP 4-monooxygenase (PdcA), *p*-benzoquinone (BQ) reductase (PdcB), hydroxyquinol (BT) 1,2-dioxygenase (PdcC), maleylacetate (MA) reductase (PdcF), 4-hydroxymuconic semialdehyde (4-HS) dehydrogenase (PdcG), and hydroquinone (HQ) 1,2-dioxygenase (PdcDE). Four genes (*pdcDEFG*) were expressed in *E. coli *and the purified *pdcDE*, *pdcG *and *pdcF *gene products were shown to convert HQ to 4-HS, 4-HS to MA and MA to β-ketoadipate respectively by *in vitro *activity assays.

**Conclusions:**

The cloning, sequencing, and characterization of these genes along with the functional PNP degradation studies identified 4-NC, HQ, 4-HS, and MA as intermediates in the degradation pathway of PNP by *Pseudomonas *sp.1-7. This is the first conclusive report for both 4-NC and HQ- mediated degradation of PNP by one microorganism.

## Background

*para*-Nitrophenol (PNP) is a common environmental pollutant owing to its wide application in pharmaceuticals, explosives, dyes and agrochemicals. PNP also accumulates in the soil due to the hydrolysis of organophosphorus insecticides such as parathion or methyl parathion (MP) [1]. Although PNP is less toxic than MP, it is also considered a significant potential toxic contaminant [2,3] and belongs to one of 275 hazardous substances commonly found at Superfund sites [4,5].

**Figure 1:**
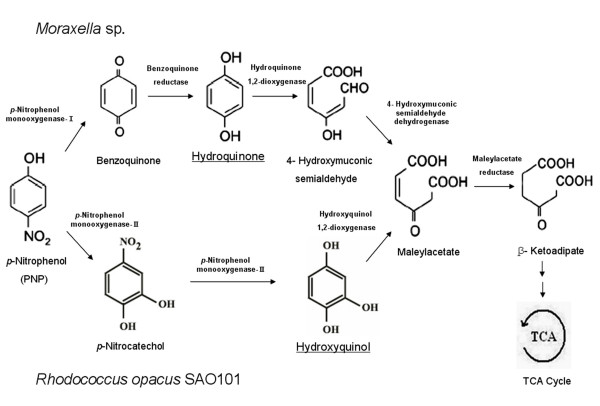
**Two alternative oxidative pathways for the metabolism of PNP**.

Many PNP-degrading bacteria have been isolated and their PNP degradation pathways studied [2,6,7]. In general, there are two alternative oxidative pathways that have been identified based on their distinct intermediates. The hydroquinone (HQ) pathway, in which PNP is degraded via HQ, is the predominant pathway in gram-negative bacteria such as *Moraxella *sp. [2] and *Pseudomonas *sp. strain WBC-3 (Figure 1, upper) [3]. The hydroxyquinol (BT) pathway is always used in gram-positive bacteria such as *Bacillus sphaericus *JS905 [7] and *Rhodococcus opacus *SAO101 [5]. PNP is degraded via 4-NC and BT in this pathway (Figure 1, lower). However, recently a gram negative organism, *Burkholderia *sp. strain SJ98, was reported to degrade PNP through the BT pathway, with no HQ pathway being detected [8]. In a gram positive organism, *Rhodococcus *sp. strain PN1, a two component PNP monooxygenase NpsA1A2 was found to catalyze PNP to both HQ and BT in the presence of ascorbic acid as a reducing reagent. However, no microbial degradation data or results from direct enzyme analyses were provided [9]. We are not aware of any reports of one bacterium being able to degrade PNP utilizing two different pathways.

Although some studies examining PNP degradation have been reported, genetic information related to the PNP degradation pathways remains limited. In the BT pathway, two enzymes were first characterized from *Rhodococcus opacus *SAO101: one was the two-component PNP monooxygenase NpcAB; the other was the one-component BT 1,2-dioxygenase NpcC. However, the other enzymes involved in this pathway have not been identified [5]. In *Arthrobacter *sp. strain JS443, another two-component monooxygenase gene *NpdA1A2 *has been identified [4]. Recently, Chauhan A *et al*. identified two lower stream genes (*pnpCD*) encoding BT 1,2-dioxygenase and maleylacetate (MA) reductase in this pathway [8]. It is worth mentioning that there are two clusters involved in PNP degradation in the gram-positive bacterium *Rhodococcus *sp. strain PN1. Within these two clusters, two kinds of two-component PNP monooxygenase genes (*nphA1A2 *and *npsA1A2*), a regulator protein gene (*npcR*) and a BT 1,2-dioxygenase gene (*npsB*) have been identified [9,10]. For the HQ pathway, the first gene cluster was obtained from *Pseudomonas *sp. strain WBC-3, and three enzymes involved in PNP degradation, PNP 4-monooxygenase (PnpA), *p*-benzoquinone (BQ) reductase (PnpB) and BT 1,2-dioxygenase (PnpG), have been characterized [3,11]. The rest of the genes in this PNP degradation gene cluster have not been identified. Recently, Shen W *et al*. identified five genes (*pnpACC1C2R*) in another gram-negative PNP-degrading bacterium, *Pseudomonas putida *DLL-E4, but the rest of the genes (*pnpBDE*) in this gene cluster were not identified [12]. To date, all the studies have focused on identifying the upper stream genes in the HQ pathway, while the knowledge of the lower stream pathway genes, especially that of the 4-HS dehydrogenase [13], remains limited.

In this study, a gram-negative bacterium *Pseudomonas *sp. 1-7, with the ability to degrade both MP and PNP, was isolated from MP-polluted activated sludge. Microbial degradation studies showed that the intermediate products were HQ and 4-NC, which indicated that both the HQ pathway and BT pathway were utilized in *Pseudomonas *sp. 1-7. Additionally, a 10.6 Kb gene cluster (*pdcEDGFCBA*) was identified from a genomic library. Genes: *pdcDE*, *pdcF *and *pdcG *were chosen to be expressed in *Escherichia coli *for characterization.

## Methods

### Strains, plasmids, and chemicals

The plasmids and bacterial strains used in this study are listed in Table 1. *Pseudomonas *sp. 1-7 was grown at 30°C in Luria Bertani (LB) medium and Burk mineral medium [14] with 1 mM MP or 0.5 mM PNP as the sole carbon and nitrogen source, respectively. *E. coli *strains were grown in LB medium at 37°C and were transformed as described [15]. The primer sequences used for PCR are listed in Additional file 1: Table S1. All reagents used in this study were purchased from Sigma Chemical (St. Louis, MO, 113 USA) and Amresco Chemical (Solon, OH 44139 USA).

**Table 1:** Bacterial strains and plasmids used in this study

Strains and plasmids	Relevant genotype or characteristic(s)	Reference or source
***Pseudomonas sp***		
Strain 1-7	methyl parathion and *p*-nitrophenol utilizer, wild type	This study
***E.coli***		
Trans10	F^-^Φ80(*lac*Z) M15 lacX74hsdR(r_K_^-^m_K_^+^) recA1398endA1tonA	TransGen
BL21(DE3)	F^- ^*ompT hsdS *(rB^- ^mB^-^) *gal dcm lacY1*(DE3)	Novagen
**Plasmids**		
pET30a	Km^r^, Expression vector	Novagen
pET22b	Amp^r^, Expression vector	Novagen
pET2230	Amp^r^, Expression vector	This study
pEASY-T3	Amp^r^, Cloning vector	TransGen
pET30- *pdcF*	*Bam*HI-*Hin*dIII fragment containing *pdcF *inserted into pET30a	This study
pET30- *pdcG*	*Bam*HI-*Xho*I fragment containing *pdcG *inserted into pET30a	This study
pET30- *pdcD*	*Bam*HI-*Xho*I fragment containing *pdcD *inserted into pET30a	This study
pET2230- *pdcE*	*Bam*HI-*Xho*I fragment containing *pdcE *inserted into pET2230	This study

### Isolation of *Pseudomonas *degrading MP and PNP

Activated sludge (0.5 *g*) collected from a pesticide factory (Tianjin, China) was cultured overnight at 30°C in 100 ml liquid Burk medium, before being diluted and spread on solid Burk medium containing 0.1% (v/v) MP pesticide and incubated at 30°C. The positive strain able to degrade MP produced a visible hydrolysis halo around the colonies on the plate. Positive colonies were inoculated in liquid Burk medium containing 0.1% (v/v) MP pesticide and cultured overnight at 30°C. MP degradation to PNP was assessed by monitoring whether the medium changed color from colorless to yellow. Further incubation for 2 days was used to test whether this was followed by PNP degradation, confirmed by a subsequent color change from yellow to colorless. Finally, the ability of this bacterium to degrade MP and PNP was confirmed by a second inoculation on a Burk agar plate containing 0.1% (v/v) MP [16].

### Extraction of the intermediates from culture

After the cultures had reached late log-phase in LB medium supplemented with 0.5 mM PNP, bacteria were harvested and washed in Burk medium by centrifugation. The bacteria were then incubated as concentrated cell suspensions (optical density of 1.5 at 600 nm) in Burk medium containing 1.5 mM PNP. Samples were collected at different time points, centrifuged, and aromatic compounds were extracted from the cell-free supernatants as described by Samanta *et al *[17].

### Characterization of intermediate compounds by HPLC and MS

Identification and quantification of intermediates was performed based on their UV-visible spectra, MS spectra and by chromatographic comparison with standards. The HPLC system consisted of an Agilent 1100 model G1312A binary pump, a model G1330B autosampler and a model G1315B DAD (Agilent Technologies, Inc., Wilmington, DE) equipped with a C18 reversed phase column (5 μm; 250 × 4.6 mm; SunFire) using a column temperature of 30°C. The mobile phase was 30% methanol (pH 3.0) at a flow rate of 0.5 ml min^-1^. PNP, HQ and 4-NC were all detected in the range 220-400 nm. Under these conditions, authentic PNP, HQ, and 4-NC had retention times of 75, 10.5 and 45 min, respectively.

MS spectra of the intermediate compounds were obtained by the following procedure: a mass selective detector (Agilent, 6430, Ion Trap) was equipped with an ESI using a cone voltage of 25 V and a capillary voltage of 3.5 kV for negative ionization of the analytes (ESI-mode). The dry nitrogen was heated to 325°C and the drying gas flow was 8 l min^-1^. Data were acquired in the negative scan mode in the range 30-500 Da. The mass of each compound was calculated from its peak area.

### Construction of a genomic DNA library

All DNA isolation and cloning procedures were carried out essentially as described by Sambrook *et al*. [15]. Construction of the fosmid library strictly followed the protocol of the CopyControl™ HTP Fosmid Library Production Kit of EPI (Epicentre Biotechnologies, Madison, WI, USA).

### Cloning of the genes involved in PNP degradation

The fosmid library was screened for the positive strains that contained the genes involved in PNP degradation using a PCR-based library screening method. The primers (Ps-F and Ps-R) (Additional file 1: Table S1) were designed based on a conserved region which was identified by comparing the amino acid sequences of available BT dioxygenase gene sequences.

### Sequence analysis

The nucleotide sequence of the positive clone was determined by the National Key Facility Open Laboratory of the Chinese Academy of Agricultural Sciences (Beijing, China). The sequences were assembled using the Contig Express program of the Vector NTI suite 7.0 (InforMax, Frederick, MD, USA). Open reading frames (ORFs) in the assembled sequence were analyzed by the ORF finder tool [18], and deduced amino acid sequences were examined by BLASTP in NCBI [19]. The potential signal peptides and hydrolytic domains of the identified genes were predicted using SignalP 3.0 (http://www.cbs.dtu.dk/services/SignalP). Multiple alignments between protein sequences were performed using ClustalW1.83.

### Expression in *E. coli *of genes involved in PNP degradation

Four genes were selected for expression in *E. coli*. Genes (*pdcDEFG*) were amplified by PCR from the positive clones, inserted into expression vectors pET30a (Novagen) or pET2230, and transformed into the expression host *E. coli *BL21 (DE3), respectively. The primers with their restriction sites are shown in Additional file 1: Table S1. The backbone and the multiple cloning sites of pET2230 originated from pET22b and pET30a, respectively. All positive colonies harboring the corresponding gene were confirmed by DNA sequencing. All host cells harboring the recombinant vectors were grown in LB at 37°C to an OD_600 _of 0.6 and then induced by the addition of IPTG (0.4 mM final concentration) and incubation at 16°C for 16 h to yield the recombined proteins with fused His_6 _tags.

### Purification of recombinant proteins

*E. coli *BL21 (DE3) cells harboring the expression plasmid of interest were harvested by centrifugation and resuspended in 20 mM Tris-HCl buffer (pH 8.0). The crude cell extracts were prepared by sonication [20]. All His-tagged recombined proteins (His_6_-PdcF, His_6_-PdcG and His_6_-PdcDE) were purified from the corresponding *E. coli *crude cell extract using Ni-nitrilotriacetic acid agarose (Ni^2+^-NTA) (Qiagen, Valencia, CA, USA) according to the manufacturer's protocol. The purified proteins were characterized by sodium dodecyl sulfate-polyacrylamide gel electrophoresis (SDS-PAGE).

### Enzymatic assays

The enzyme assays are described in the Additional file 1 (Methods of Enzyme Assays). All assays, where applicable, were performed using cell extracts prepared from non-induced BL21 (DE3) cells that harbored the corresponding recombinant vector and from BL21 (DE3) cells that harbored the non-recombinant expression vector as the negative controls.

### GenBank accession number

The nucleotide sequences of the *Pseudomonas *sp. 1-7 16S rDNA and the PNP degradation gene cluster were deposited in the GenBank database [GenBank FJ821774 and GenBank FJ821777, respectively].

## Results

### Isolation of *Pseudomonas *sp. 1-7

Strain 1-7, capable of degrading both MP and PNP and collected from a pesticide factory in Tianjin, China, was identified as a *Pseudomonas *sp. by 16S rDNA analysis, which sequence has been deposited in the Agricultural Culture Collection of China (ACCC), with collection number [ACCC 05510] [16]. When *Pseudomonas *sp. 1-7 was cultured in Burk medium containing 0.1% (v/v) MP pesticide, the color of the culture changed to yellow from colorless, indicating that the MP had been hydrolyzed to PNP. After incubation for a further 2 days, the color reverted to colorless, indicating PNP degradation. Moreover, this strain exhibited the same phenomenon on a culture plate containing 0.1% (v/v) MP pesticide: generation of a distinct hydrolysis halo, the color of which first turned yellow and then became colorless. *Pseudomonas sp*. 1-7 was thus able to degrade both MP and PNP.

In former studies, the full-length of methyl parathion hydrolase gene *ophc3 *from this bacterium was cloned by constructing genomic library. The gene *ophc3 *was expressed in *E*. *coli *and recombinant methyl parathion hydrolase OPHC3 was purified and the enzymatic properties were studied [16].

### Strain 1-7 degraded PNP utilizing both HQ and BT pathways

**Figure 2:**
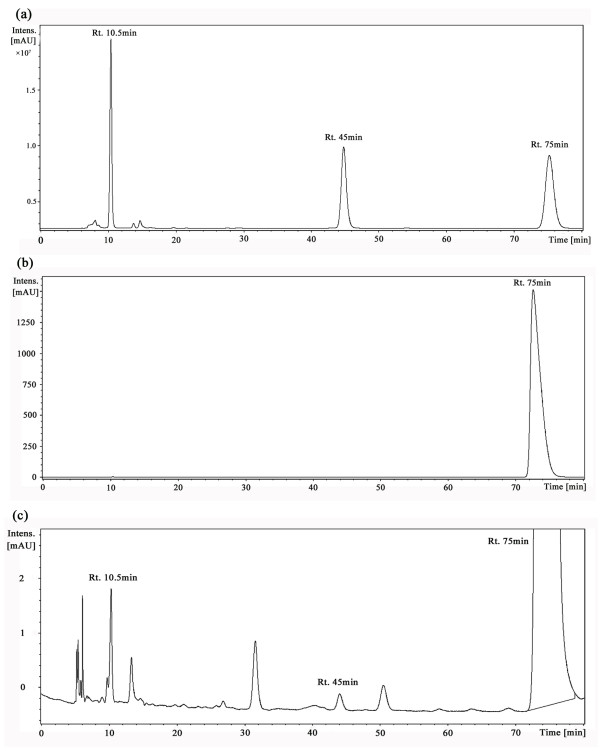
**HPLC analyses of supernatants of *Preudomonas *sp. 1-7 grown on PNP**. (**a**) HPLC chemical standards: authentic PNP, HQ and 4-NC had retention times of 75, 10.5 and 45 min, respectively; HPLC analysis of cell-free supernatants at (**b**) 0 h and (**c**) 3.5 h.

To determine how *Pseudomonas *sp. 1-7 degraded PNP, the reaction intermediates were analyzed by HPLC. The analyses yielded three distinct peaks with retention times of 10.5 min, 45 min, and 75 min in samples drawn at 0-3.5 h intervals. These retention times corresponded with those of the standard compounds HQ, 4-NC and PNP, respectively (Figure 2). In addition, the 220-400 nm absorption spectra of all the detected peaks corresponded with those of the standard compounds (Additional file 1: Figure S1). The HPLC studies thus confirmed the presence of PNP, 4-NC and HQ in the culture medium.

**Figure 3:**
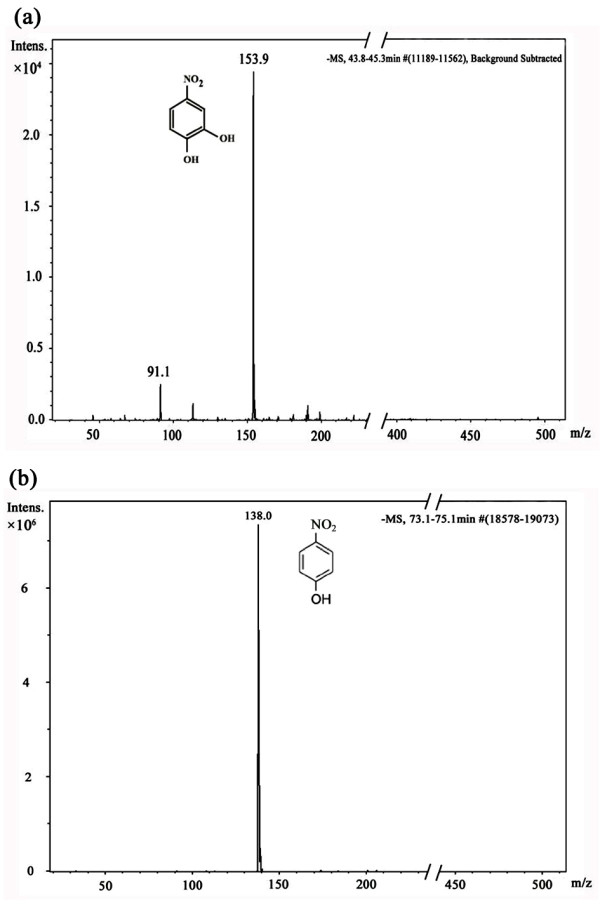
**LC-MS analyses of supernatants of *Pseudomonas *sp. 1-7 grown on PNP**. Mass of the intermediates identified in the peaks with retention times of 45 min (**a**) and of 75 min (**b**) in the sample extracted after 3.5 h.

The LC-MS analyses of the 3.5 h HPLC samples showed the two peaks with the retention times of 45 min and 75 min as having molecular ion at *m*/*z *of 153.9 and 138.0, respectively (Figure 3). These *m*/*z *results matched the standard *m*/*z *of 4-NC and PNP and confirmed the identities of the two peaks as 4-NC and PNP, respectively. However, because the nonpolar HQ molecule could not be detected by LC-MS, we were unable to confirm that the HPLC peak with the retention times of 10.5 min was, in fact, HQ.

Additionally, culture supernatants collected at various time intervals showed a sharp depletion of PNP within 3.5 h, and clearly demonstrated the accumulation of HQ and 4-NC from 3.5 h onward. The maximum amount of 4-NC was detected at 3.5 h, and the maximum amount of HQ at 30 min (Additional file 1: Figure S2).

These results identified both HQ and 4-NC as intermediates in the degradation of PNP by strain 1-7. Being intermediates in the HQ and BT pathways, respectively, both of the two PNP degradation pathways are therefore utilized in PNP mineralization by strain 1-7.

### Cloning of genes involved in PNP degradation

**Figure 4:**
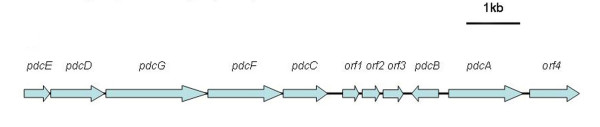
**Organization of the putative ORFs in *Pseudomonas *sp. 1-7**. Organization of putative ORFs in the 10.6-kb DNA fragment. The arrows indicate the size and direction of each ORF.

Two positive clones (4-2 M and 4-8 G) were obtained by PCR-based screening of the genomic library of strain 1-7, and a 10.6 kb fragment in 4-2 M containing 11 complete ORFs (*pdcABCDEFG*, *orf1*, *orf2*, *orf3*, *orf4*) was cloned. Their annotations were determined from BLAST analysis, and the ORF organization is shown in Figure 4. Genes *pdcABCDEFG *showed a high similarity with the reported PNP degradation cluster (*pnpABCDEFG*) from *Pseudomonas *sp. strain WBC-3 [3], and the proteins PdcABCDEFG had no potential signal peptides as determined by SignalP 3.0.

### Expression and purification of PdcF, PdcG and PdcDE

**Figure 5:**
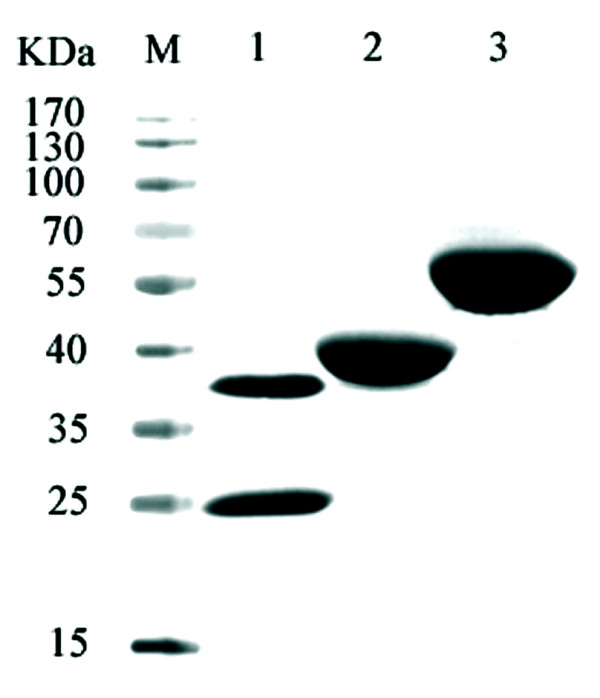
**SDS-PAGE of purified recombinant His_6_-PdcDE, His_6_-PdcF and His_6_-PdcG**. Lane M: molecular mass standards (sizes in kDa are shown on the left); lane 1: purified His_6_-PdcDE; lane 2: purified His_6_-PdcF; lane 3: purified His_6_-PdcG.

To characterize the enzymes involved in PNP degradation, four genes (*pdcDEFG*) were expressed in *E. coli *BL21 (DE3). After purification by Ni^2+^-NTA affinity chromatography, the proteins His_6_-PdcF, His_6_-PdcG, His _6_-PdcD and His _6_-PdcE had been purified to apparent homogeneity by SDS-PAGE analysis. Their molecular masses were 37 kDa, 52 kDa, 38 kDa and 18 kDa, respectively (Figure 5), being consistent with the calculated molecular masses of these proteins.

### Enzymatic assays of HQ 1,2-dioxygenase activity

**Figure 6:**
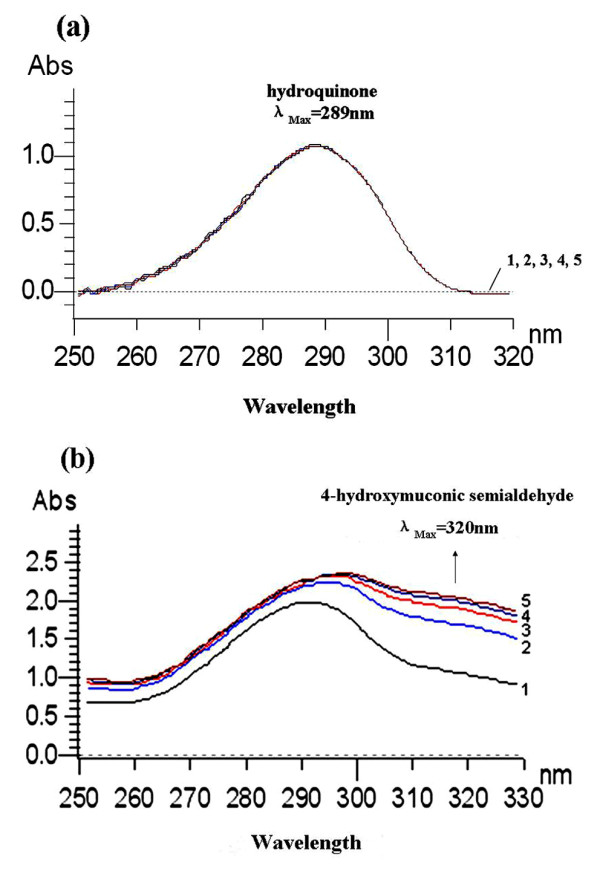
**Enzyme activity assay of PdcDE**. (**a**) Absorbance readings from 250 nm to 320 nm in the absence of His_6_-PdcDE; (**b**) Spectral changes during rapid oxidation of HQ by purified His_6_-PdcDE. The spectra were recorded a total of five times over a five minute period (marked 1-5). The arrows indicate the direction of spectral changes.

HQ 1,2-dioxygenase, being the third enzyme of the HQ pathway, catalyzes the ring cleavage reaction of HQ to 4-HS [21]. Two genes (*pdcD *and *pdcE*) were cloned into the expression vectors pET-30a and pET-2230, respectively, and PdcD and PdcE were co-expressed in *E*. *coli *BL21 (DE3) to allow endogenous assembly of the active HQ 1,2-dioxygenase. Spectrophotometric analysis of HQ 1,2-dioxygenase (His_6_-PdcDE) activity showed a spectral change from 290 nm to 320 nm during the oxidation of HQ by His_6_-PdcDE (Figure 6b), there being no spectral changes in the negative controls (Figure 6a). These results indicated that His_6_-PdcDE catalyzed the ring cleavage reaction of HQ to 4-HS.

His_6_-PdcDE was active over a temperature range of 20-70°C, with an optimal activity at 40°C, and from pH 3.0-10.0 with an optimum activity at pH 6.0 (Table 2, Additional file 1: Figure S3a, S3c). Further, the purified enzyme retained 35% activity after 20 min at 60°C, 20% activity after 30 min at pH 3.0 and 60% activity after 30 min at pH 10.0 (Additional file 1: Figure S3b, S3d). The influence of different metal ions, EDTA and SDS on enzyme activity is shown in Table 3.

**Table 2:** Biochemical properties of the three enzymes

Enzyme	Temperature range(°C)	Optimal temperature	**Thermal Stability**^①^	pH range	Optimal pH	**Acid stability**^②^	**Alkali Stability**^③^	Specific activity
**PdcDE**	20-70	40°C	35%	3.0-10.0	6.0	20%	60%	ND^④^
**PdcG**	20-70	50°C	65%	5.0-10.0	8.0	18%	75%	0.44 U/mg
**PdcF**	20-70	40°C	10%	5.0-9.0	7.0	20%	58%	446.97 U/mg

①Relative activity of purified protein when it was treated in 60°C for 20 min;

②Relative activity of purified protein when it was treated in pH 3.0 for 30 min;

③Relative activity of purified protein when it was treated in pH 10.0 for 30 min;

④Not detectedEach value represents the mean of at least three independent replicates.

**Table 3:** Effect of various metal ions and chemical agent on the activity of the three enzymes

Metal ion or chemical agent (5 mM)		Relative activity (%)	
	
	PdcDE	PdcG	PdcF
**No addition**	100	100	100
**K^+ ^(KCl)**	113.04 ± 10.80	95.79 ± 16.49	129.00 ± 27.32
**Na^+ ^(NaCl)**	113.42 ± 2.27	88.22 ± 17.76	123.91 ± 25.82
**Ba^2+ ^(BaCl_2_)**	99.19 ± 6.29	123.34 ± 7.79	129.02 ± 6.46
**Mg^2+ ^(MgCl_2_)**	95.41 ± 5.96	138.06 ± 8.46	129.79 ± 18.11
**Zn^2+ ^(ZnCl_2_)**	87.44 ± 8.68	145.95 ± 5.13	21.44 ± 3.71
**Cu^2+ ^(CuCl_2_)**	22.46 ± 6.83	110.18 ± 11.17	59.23 ± 12.57
**Ni^2+ ^(NiCl_2_)**	111.05 ± 2.61	183.93 ± 30.68	35.25 ± 16.67
**Co^2+ ^(CoCl_2_)**	104.15 ± 6.79	147.08 ± 17.51	79.14 ± 13.21
**Mn^2+ ^(MnCl_2_)**	77.45 ± 2.93	186.12 ± 9.99	136.59 ± 3.65
**Cd^2^+ (CdSO_4_)**	63.24 ± 3.61	58.93 ± 3.88	39.52 ± 7.01
**Fe^2+ ^(FeCl_2_)**	82.13 ± 13.46	39.47 ± 9.49	118.90 ± 21.53
**Fe^3+ ^(FeCl_3_)**	78.33 ± 10.74	187.37 ± 15.37	134.89 ± 28.19
**EDTA**	62.44 ± 3.90	83.17 ± 8.32	112.93 ± 40.43
**SDS**	97.47 ± 1.65	81.58 ± 24.05	136.59 ± 3.66

Each value represents the mean of at least three independent replicates.

### Enzymatic assays of 4-HS dehydrogenase activity

**Figure 7:**
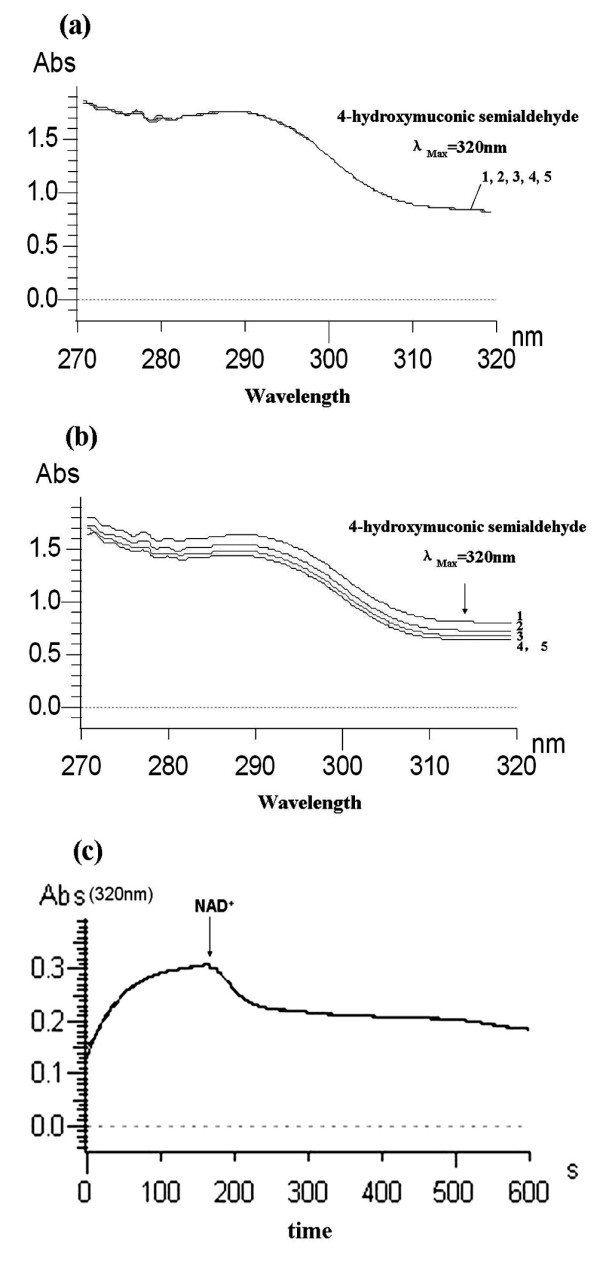
**Enzyme activity assay of PdcG**. (**a**) Absorbance from 270 nm to 320 nm in the absence of His_6_-PdcG; (**b**) Spectral changes during oxidation of 4-HS by His_6_-PdcDE. The spectra were recorded a total of five times over a five minute period (marked 1-5). The arrow indicates the direction of spectral changes. (**c**) Spectral changes at 320 nm during metabolism of HQ by purified His_6_-PdcDE and oxidation of 4-HS by purified His_6_-PdcG. The arrow indicates when NAD^+ ^was added.

The catalysis of 4-HS to MA by 4-HS dehydrogenase (His_6_-PdcG) was determined by monitoring the spectral changes at 320 nm. During this enzyme assay, the absorbance at 320 nm became progressively lower after purified His_6_-PdcG had been added to the reaction mixture in the presence of NAD^+ ^(Figure 7b). There was no disappearance of 4-HS in the negative controls (Figure 7a). His_6_-PdcG thus catalyzed the oxidation of 4-HS to MA, confirming that PdcG was the enzyme downstream of PdcDE in the PNP degradation pathway in strain 1-7.

The specific activity of PdcG was calculated to be 0.44 Umg^-1 ^(Table 2). PdcG was active over a temperature range of 20-70°C with an optimal activity at 50°C, and over a pH range of 5.0-10.0 with an optimum activity at pH 8.0 (Additional file 1: Figure S4a, S4c). Further, the purified enzyme retained 65% activity after 20 min at 60°C, 18% activity after 30 min at pH 3.0, and 75% activity after 30 min at pH 10.0 (Additional file 1: Figure S4b, S4d). The influence of different metal ions, EDTA and SDS is shown in Table 3.

### Co-action of PdcDE and PdcG

Because PdcG was able to metabolize the product of PdcDE, the activities of both His_6_-PdcDE and His_6_-PdcG were assayed in one reaction mixture with HQ as the substrate. This was done spectrophotometrically by following the change of absorbance at 320 nm. At the beginning of the reaction, the absorbance at 320 nm rose continuously (Figure 7c), while no rising curve was observed in the negative control (data not shown). This indicated that 4-HS was generated in the reaction mixture containing both enzymes. After about 180 seconds, the absorbance plateaued, suggesting that the generation of 4-HS had reached a limit. NAD^+ ^(the cofactor of PdcG) was then added to the reaction mixture to a final concentration of 0.05 mM to activate His_6_-PdcG. Upon addition of NAD^+^, the absorbance at 320 nm immediately decreased rapidly, and then leveled off. However, no such results were observed when His_6_-PdcG was omitted from the reaction or when His_6_-PdcDE was incubated with a crude cell extract of the non-induced BL21 strain that harbored *pdcF *instead of His_6_-PdcG (data not shown). This confirmed that 4-HS was the product of His_6_-PdcDE acting on HQ, and that 4-HS was the substrate of the enzyme His_6_-PdcG.

### Enzymatic assays of MA reductase activity

**Figure 8:**
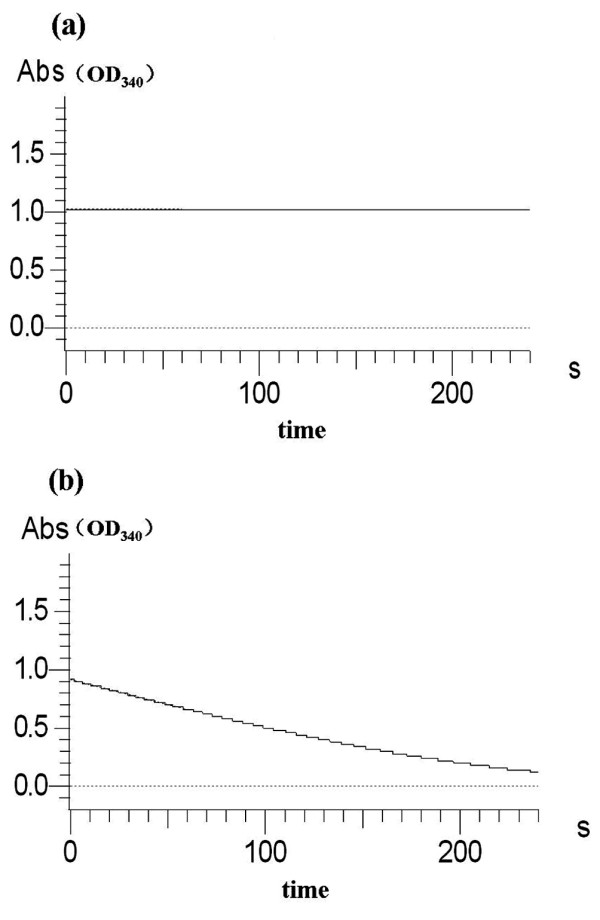
**Enzyme activity assay of PdcF**. (**a**) Absorbance at 340 nm in the absence of His_6_-PdcF; (**b**) Absorbance at 340 nm during oxidation of NADH by His_6_-PdcF.

MA reductase is the common enzyme of the two PNP degradation pathways and uses NADH as a cofactor [22]. In the MA reductase (His_6_-PdcF) assay, the decrease in absorption at 340 nm was used to monitor the conversion of NADH to NAD^+ ^(ε340 NADH = 6.3 mM-1 cm-1), which conversion reflects the activity of His_6_-PdcF. When purified His_6_-PdcF was added to the assay mixture, there was significant oxidation of NADH (Figure 8a). However, no oxidation of NADH was observed when His_6_-PdcF was omitted from the reaction (Figure 8b). Thus, PdcF reduced MA to β-ketoadipate with NADH as a cofactor.

His_6_-PdcF was active over a temperature range of 20-70°C with an optimal activity at 40°C, and over a pH range of 5.0-9.0 with an optimum activity at pH 7.0 (Table 2, Additional file 1: Figure S5a, S5c). Its specific activity was calculated to be 446.97 Umg^-1^. Further, the purified enzyme retained 10% activity after 20 min at 60°C, 20% activity after 30 min at pH 3.0, and 58% activity after 30 min at pH 10.0 (Additional file 1: Figure S5b, S5d). The influence of different metal ions, EDTA and SDS is shown in Table 3.

## Discussion

*Pseudomonas *sp.1-7, a gram-negative bacterium isolated from MP-polluted activated sludge, was able to catabolize both MP and its initial hydrolysis product PNP. Using HPLC and LC-MS, we demonstrated that strain 1-7 degraded PNP through two different pathways, the HQ pathway and the BT pathway. A gene cluster *pdcABCDEFG *involved in PNP degradation was identified in *Pseudomonas *sp.1-7. Genes *pdcABDEFG *were involved in the HQ pathway, and genes *pdcCG *were involved in the BT pathway. The BT pathway also needs a two-component PNP monooxygenase (Figure 1) to catalyze PNP to 4-NC and BT [5]; however, we did not find the relevant PNP monooxygenase in the gene cluster. We speculate that the monooxygenase PdcA in the HQ pathway may have two functions, catalyzing PNP to both BQ and 4-NC. This is supported by recent reports indicating that the HQ pathway monooxygenase has the ability to catalyze 4-NC to BT, normally thought to be the work of the BT pathway monooxygenase [11]. This suggests that the HQ pathway monooxygenase could be substituted for the BT pathway monooxygenase in the process of PNP degradation. In future studies, we will identify whether there are BT pathway-specific PNP monooxygenase genes, or whether the HQ pathway monooxygenase is a bi-functional enzyme in strain 1-7.

We also identified three enzymes (PdcDE, PdcF and PdcG) in the HQ pathway. PdcDE was a two-component dioxygenase and catalyzed HQ to 4-HS. PdcG was a 4-HS dehydrogenase that catalyzed 4-HS to MA. PdcF was a MA reductase which transformed MA to β-ketoadipate. All three enzymes performed optimally at temperatures of 40-50°C, and at nearly neutral pH (pH 6.0-8.0). Regarding stability, only PdcG has a better thermal stability at 60°C (65% retention of activity after 20 min exposure) than the other two enzymes (10% to 35% retention). All of the enzymes had better alkali stability at pH 10.0 (58% to 75% retention of activity after 30 min exposure) than acid stability at pH 3.0 (18% to 20% retention).

The HQ dioxygenase gene has been identified in other bacteria [12,21], but little is known about the properties of its corresponding enzyme. Our research on the enzyme (PdcDE) will hopefully contribute to our understanding. Of the two, the MA reductase PdcF was the more active enzyme, with a specific activity of 446.97 Umg^-1 ^as opposed to 13.33 Umg^-1^. It is also the first time that a 4-HS dehydrogenase (PdcG) has been extensively characterized.

## Conclusions

*Pseudomonas *sp.1-7, with the capability of degrading MP and PNP, was isolated from MP-polluted activated sludge. The bacterium utilized two pathways for PNP degradation, the HQ pathway and the BT pathway. Three enzymes (PdcDE, PdcF and PdcG) in the HQ pathway were expressed, purified, and characterized. Our research will pave the way for a better understanding of the PNP degradation pathway in gram-negative bacteria.

## Abbreviations

4-HS: 4-hydroxymuconic semialdehyde; 4-NC: 4-Nitrocatechol; BT: Hydroxyquinol; BQ: *p*-benzoquinone; HQ: Hydroquinone; MP: Methyl parathion; MA: Maleylacetate; PNP: *para*-Nitrophenol.

## Authors' contributions

NFW designed the experiment and revised the manuscript. SYZ carried out most of molecular genetic studies and drafted the manuscript. WS and LX participated part of experiments. XYC and JT conceived of the study, participated in its design and coordination and helped to draft the manuscript. YLF and XMZ revised the manuscript and give many important suggestions. All authors read and approved the final manuscript.

## Supplementary Material

Additional file 1 **This section mainly involves the methods of the enzyme assays and some figure data about the enzyme charactication**. Additionally, this file also includes a table about the primers used in this study, a figure reflects the concentration changes of the substrate and of the two intermediates in the course of the PNP degradation and another figure about the specific absorbs curved line which reflects the detected peak by HPLC [13,21,22]. Click here for file
