# Supplementary material for: Identification of the para-nitrophenol catabolic pathway, and characterization of three enzymes involved in the hydroquinone pathway, in pseudomonas sp. 1-7
Source: BMC Microbiol. 2012 Mar 2;12:27. doi: 10.1186/1471-2180-12-27 (PMC3324391; doi:10.1186/1471-2180-12-27)
Supplement: Additional file 1 — This section mainly involves the methods of the enzyme assays and some figure data about the enzyme charactication. Additionally, this file also includes a table about the primers used in this study, a figure reflects the concentration changes of the substrate and of the two intermediates in the course of the PNP degradation and another figure about the specific absorbs curved line which reflects the detected peak by HPLC [13,21,22]. [file 1471-2180-12-27-S1.DOC]

**Methods of enzyme assays**

**(ⅰ) Hydroquinone 1, 2-dioxygenase.** The enzyme activity assay was performed as described [13, 21], and the activity of His_6_- PdcDE was determined spectrophotometrically by monitoring the formation of 4-HS at 290 nm to 320 nm. The reaction mixture contained 20 mM phosphate buffer (pH 6.0), 50 μl of purified protein (0.75 mg/ml) and 0.5 mM HQ in a final volume of 1.0 ml. The reaction was started by the addition of 50 μl of purified enzyme. Following incubation at 40°C for 1 min, the reaction was terminated through the addition of 1.0 ml of ethanol. The mixtures were centrifuged, and the absorbance of the resulting supernatants was measured at 320 nm by use of Hitachi 3100 spectrophotometer (Hitachi, Japan).

The absorbance changes at 320 nm were subsequently used to assay the enzyme's other characteristics. Its optimal pH at 40°C was determined using buffers ranging in pH from 3.0 to 12.0. The following buffers were used; 20 mM disodium hydrogen phosphate-citric acid buffer (pH 3.0–8.0), 50 mM Tris–HCl buffer (pH 8.0–10.0), and 20 mM glycine–NaOH (pH 10.0–12.0). To test its pH stability, 100 μl of the PdcDE (0.75 mg/ml) was pre-incubated without substrate in 900 μl of different buffers with pH values from 3.0 to 12.0 for 0.5 h at 25°C, and then PdcDE activity was measured under standard conditions.

The enzyme's optimal temperature was determined by measuring its activity in 20 mM phosphate buffer (pH 6.0) at temperatures ranging from 20°C to 70°C. Its thermostability was subsequently monitored by pre-incubating the enzyme without substrate in 20 mM phosphate buffer (pH 6.0) for 20 min at 50, 60, and 70°C. PdcDE activity was then measured under standard conditions.

The effects of different metal ions and chemical reagents on enzymatic activity were assessed in 20 mM phosphate buffer (pH 6.0) at 40°C. The reactions contained 5 mM NaCl, KCl, BaCl_2_, FeCl_2_, FeCl_3_, CoCl_2_, CuCl_2_, NiCl_2_, MgCl_2_, MnCl_2_, ZnCl_2_, CdSO_4_, EDTA and 0.1% (v/v) sodium dodecyl sulphate (SDS).

**(ⅱ) 4-hydroxymuconic semialdehyde dehydrogenase.** The activity of 4-HS dehydrogenase was determined spectrophotometrically by monitoring the deterioration of 4-HS at 290 nm to 320 nm. The reaction mixture contained 500 μl solution, which was the product of degradation by the HQ 1,2-dioxygenase, as the substrate, and 445 μl 20 mM phosphate buffer (pH 7.0), 5 μl 10 mM NAD^+^, and 50 μl of purified protein (0.75 mg/ml) in a final volume of 1.0 ml. The assay was initiated by the addition of purified protein. Following incubation at 50°C for 2 min, the reaction was terminated through the addition of 1.0 ml of ethanol. The mixtures were centrifuged, and the absorbance of the resulting supernatants was measured at 260 nm by use of Hitachi 3100 spectrophotometer (Hitachi, Japan). One unit (U) of enzyme activity was defined as the amount of enzyme that decreases of 1 μmol of NAD^+^ per minute at 50°C.

The decrease of NAD^+^ was subsequently used to assay the enzyme's other characteristics. Its optimal pH at 50°C was determined using buffers ranging in pH from 3.0 to 12.0. The following buffers were used; 20 mM disodium hydrogen phosphate-citric acid buffer (pH 3.0–8.0), 50 mM Tris–HCl buffer (pH 8.0–10.0), and 20 mM glycine–NaOH (pH 10.0–12.0). To test its pH stability, 100 μl of the PdcG (0.75 mg/ml) was pre-incubated without substrate in 900 μl of different buffers with pH values from 3.0 to 12.0 for 0.5 h at 25°C, and then PdcG activity was measured under standard conditions.

The enzyme's optimal temperature was determined by measuring its activity in 20 mM phosphate buffer (pH 8.0) at temperatures ranging from 20°C to 70°C. Its thermostability was subsequently monitored by pre-incubating the enzyme without substrate in 20 mM phosphate buffer (pH 8.0) for 20 min at 40, 50, and 60°C. PdcG activity was then measured under standard conditions.

The effects of different metal ions and chemical reagents on enzymatic activity were assessed in 20 mM phosphate buffer (pH 8.0) at 50°C. The reactions contained 5 mM NaCl, KCl, BaCl_2_, FeCl_2_, FeCl_3_, CoCl_2_, CuCl_2_, NiCl_2_, MgCl_2_, MnCl_2_, ZnCl_2_, CdSO_4_, EDTA and 0.1% (v/v) sodium dodecyl sulphate (SDS).

**(ⅲ) Hydroquinone 1,2-dioxygenase and 4-hydroxymuconic semialdehyde dehydrogenase.** To confirm the activity of HQ 1,2-dioxygenase and 4-HS dehydrogenase, the two enzymes were assayed in the same reaction mixture. The reaction mixture contained 885 μl 20 mM phosphate buffer (pH 7.0), 50 μl His_6_-PdcDE (0.75 mg/ml), 50 μl His_6_-PdcG (0.75 mg/ml), and 0.5 mM HQ in a final volume of 1.0 ml. The assay was initiated by addition of the substrate and changes were monitored at 340 nm. When the change in absorbance leveled off, NAD^+^ was added to the reaction mixture to a final concentration of 0.05 mM, and the changes were also monitored at 340 nm.

**(ⅳ) Maleylacetate reductase**. The activity of MA reductase was assayed according to Dayna L [22] . The activity to metabolize MA was assayed by monitoring the content change of NADH in the reaction mixture (absorbance changes at 340 nm). The reaction mixture contained 680 μl solution, which was the product of degradation by BT 1,2-dioxygenase , as the substrate, and 260 μl 20 mM phosphate buffer (pH 7.0), 10 μl 10 mg/ml NADH and 50 μl purified His_6_- PdcF (1.5 μg/ml) in a final volume of 1.0 ml. The assay was initiated by the addition of the purified protein. Following incubation at 40°C for 2 min, the reaction was terminated through the addition of 1.0 ml of ethanol. Then the mixtures were centrifuged, and the absorbance of the resulting supernatants was measured at 340 nm by use of Hitachi 3100 spectrophotometer (Hitachi, Japan). One unit (U) of enzyme activity was defined as the amount of enzyme that decreases of 1 μmol of NADH per minute at 40°C.

The decrease of NADH was subsequently used to assay the enzyme's other characteristics. Its optimal pH at 40°C was determined using buffers ranging in pH from 3.0 to 12.0. The following buffers were used; 20 mM disodium hydrogen phosphate-citric acid buffer (pH 3.0–8.0), 50 mM Tris–HCl buffer (pH 8.0–10.0), and 20 mM glycine–NaOH (pH 10.0–12.0). To test its pH stability, 100 μl of the PdcF (1.5 μg/ml) was pre-incubated without substrate in 900 μl of different buffers with pH values from 3.0 to 12.0 for 0.5 h at 25°C, and then PdcF activity was measured under standard conditions.

The enzyme's optimal temperature was determined by measuring its activity in 20 mM phosphate buffer (pH 7.0) at temperatures ranging from 20°C to 70°C. Its thermostability was subsequently monitored by pre-incubating the enzyme without substrate in 20 mM phosphate buffer (pH 7.0) for 20 min at 40, 50, and 60°C. PdcF activity was then measured under standard conditions.

The effects of different metal ions and chemical reagents on enzymatic activity were assessed in 20 mM phosphate buffer (pH 6.0) at 40°C. The reactions contained 5 mM NaCl, KCl, BaCl_2_, FeCl_2_, FeCl_3_, CoCl_2_, CuCl_2_, NiCl_2_, MgCl_2_, MnCl_2_, ZnCl_2_, CdSO_4_, EDTA and 0.1% (v/v) sodium dodecyl sulphate (SDS).

**Tables and Figures**

**Table S1. Primers used in this study**

| **Primers** | **Sequences (5’-3’)^*^** | **Target** |
| --- | --- | --- |
| **Ps-F**  **Ps-R**  **16s-F**  **16s-R**  **Mal-F**  **Mal-R**  **Hys-F**  **Hys-R**  **Dio2A-F**  **Dio2A-R**  **Dio2B-F**  **Dio2B-R** | GAGAGCACCGTGYTNGGBCCSTT  CGGGCCGTCGCTSGGDATNGGRTA  GCAGGCCTAACACATGCAA  GTTACGACTTCACCCCAGTCAT  cgcggatccATGAATCCTTTCATTTACCAAAGCC  cccaagcttTCAGGCCGGTGGCAACCCTTTC  cgcggatccATGCAAAATCAGCTCTATATCGAT  ccgctcgagTCAGCGCTTGAAGTGGGGAGCGAT  cgcggatccATGGCCATGCTTGAATCCGCTGTC  ccgctcgagCTAGAACTCGATCGGATAAGGCTT  cgcggatccATGAGTAACGCTGCAGTCAATACG  ccgctcgagTTACTTGAGACAGATCTCTGCCCA | Small fragment of *pdcC*  *16SrDNA* of strain 1-7  *pdcF*  *pdcG*  *pdcD*  *pdcE* |

^*^ Specified restriction sites are underlined.


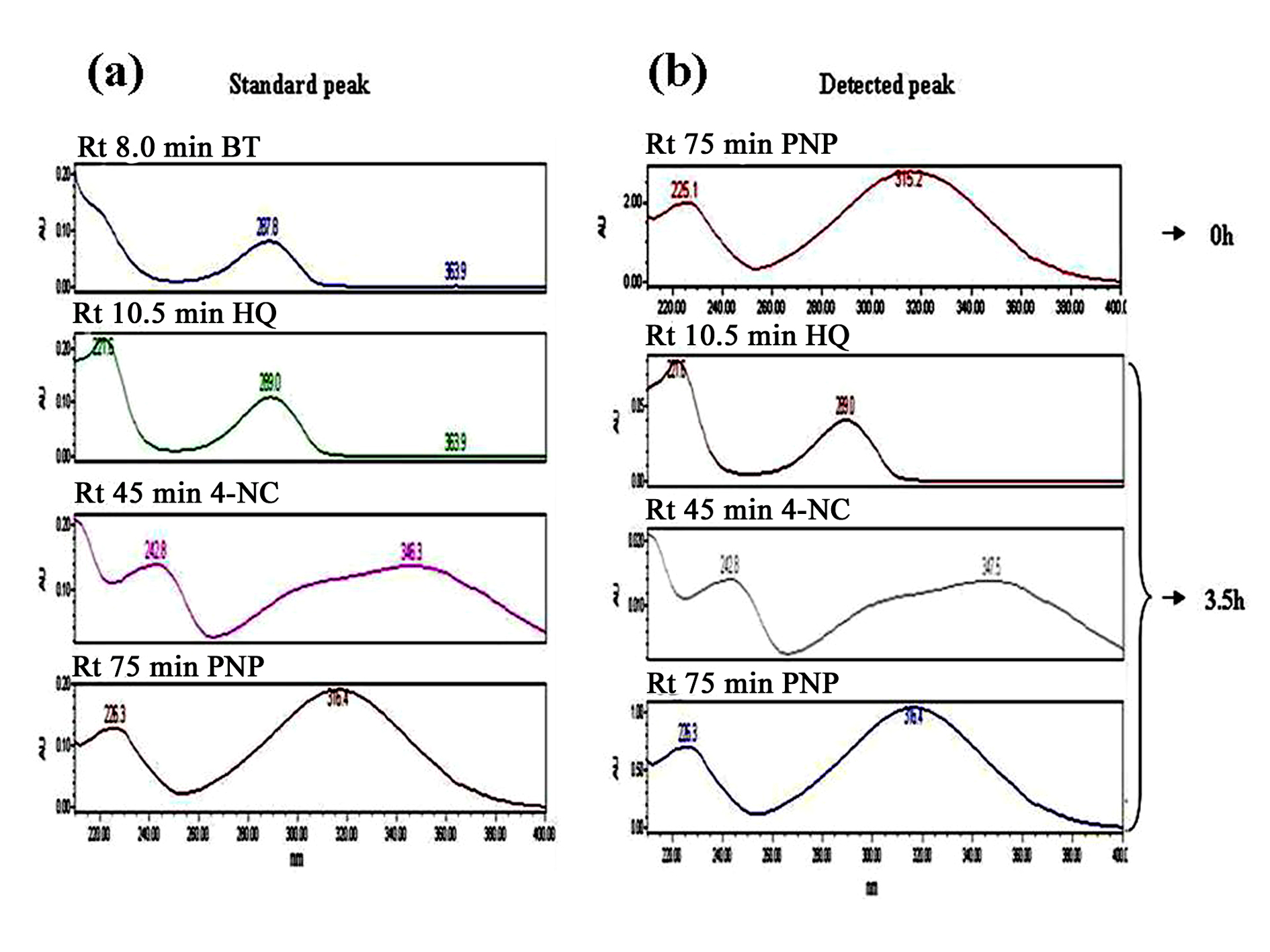


**Figure S1. HPLC analyses specific absorb curved line of (a) standard peak and (b) detected peak.**

**
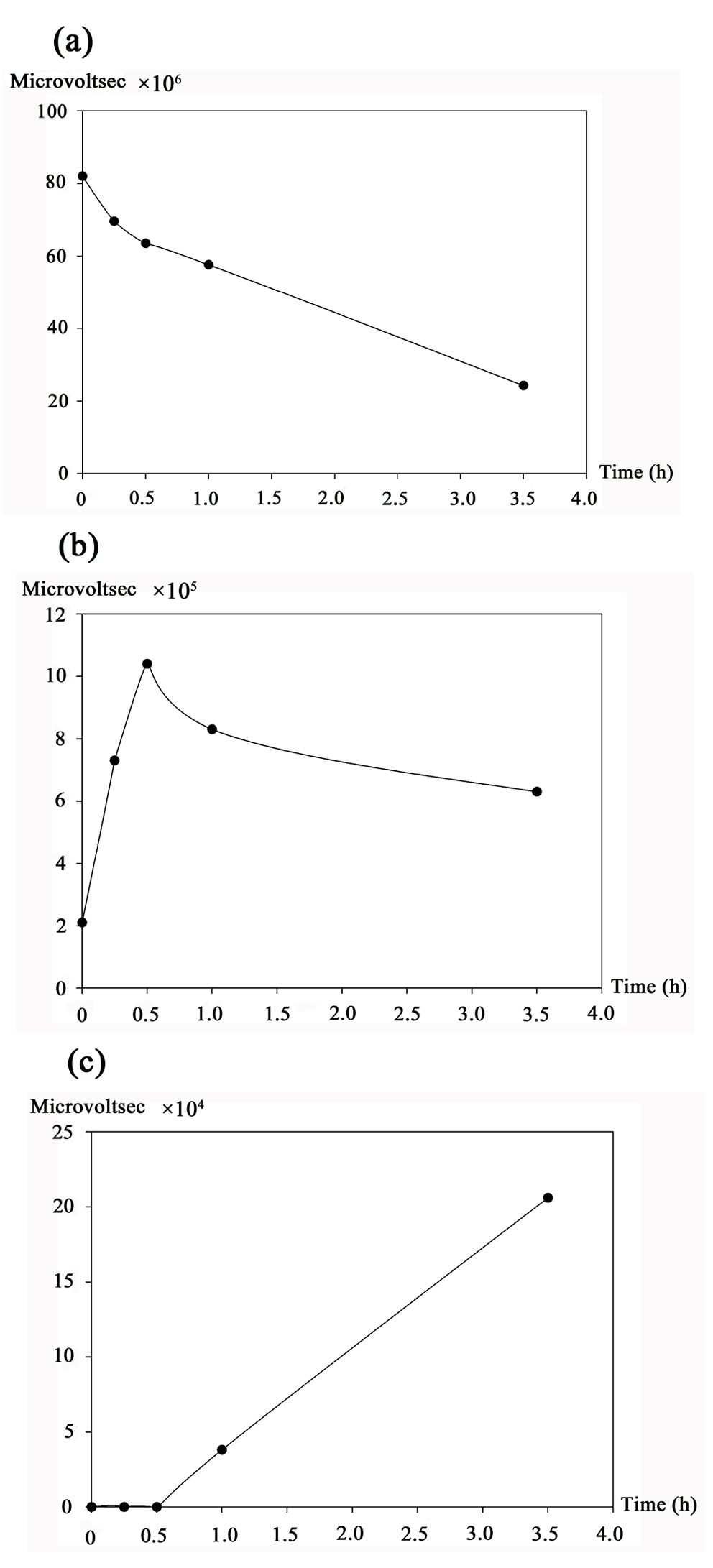
**

**Figure S2. PNP and PNP degradation intermediates changes in different time point of strain 1-7 grown on PNP.** (a) PNP, (b) HQ, (c) 4-NC.

**
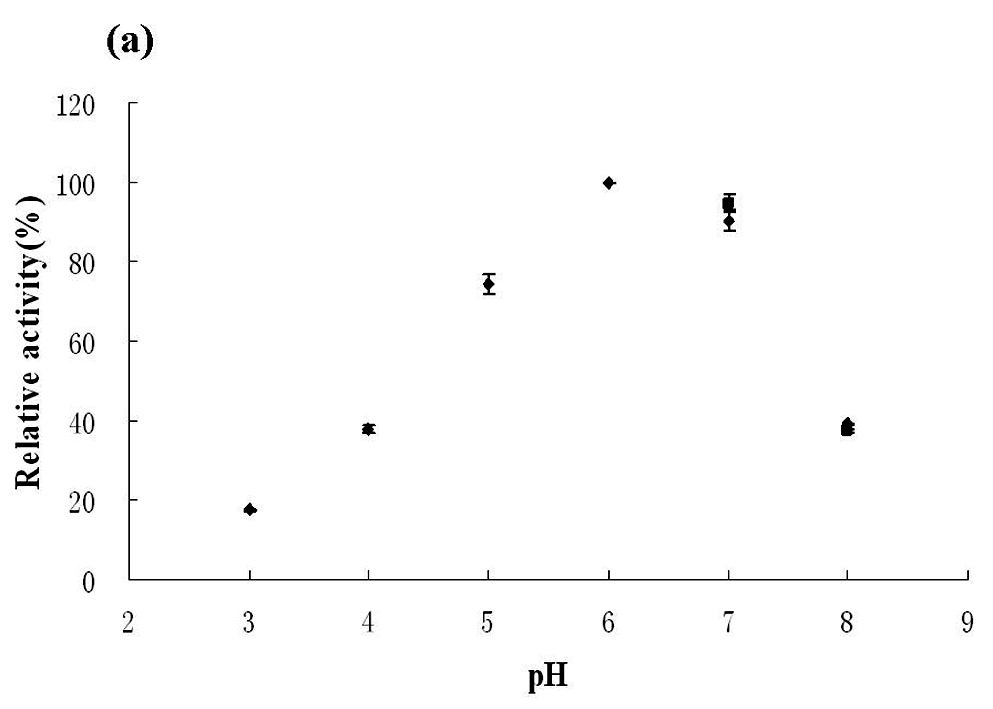
**

**
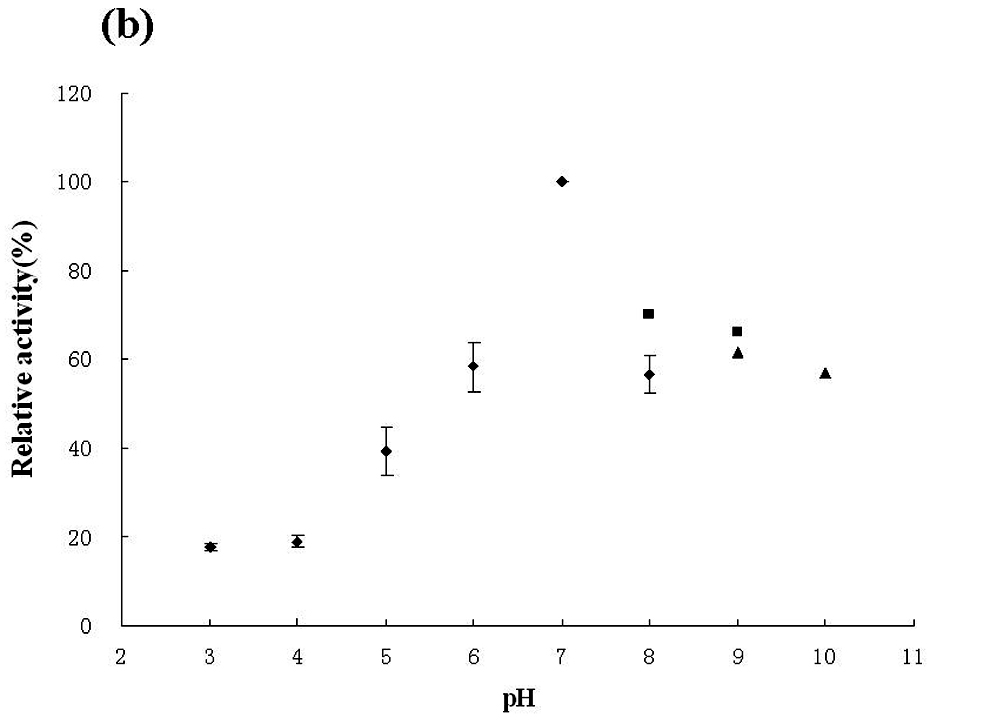
**

**
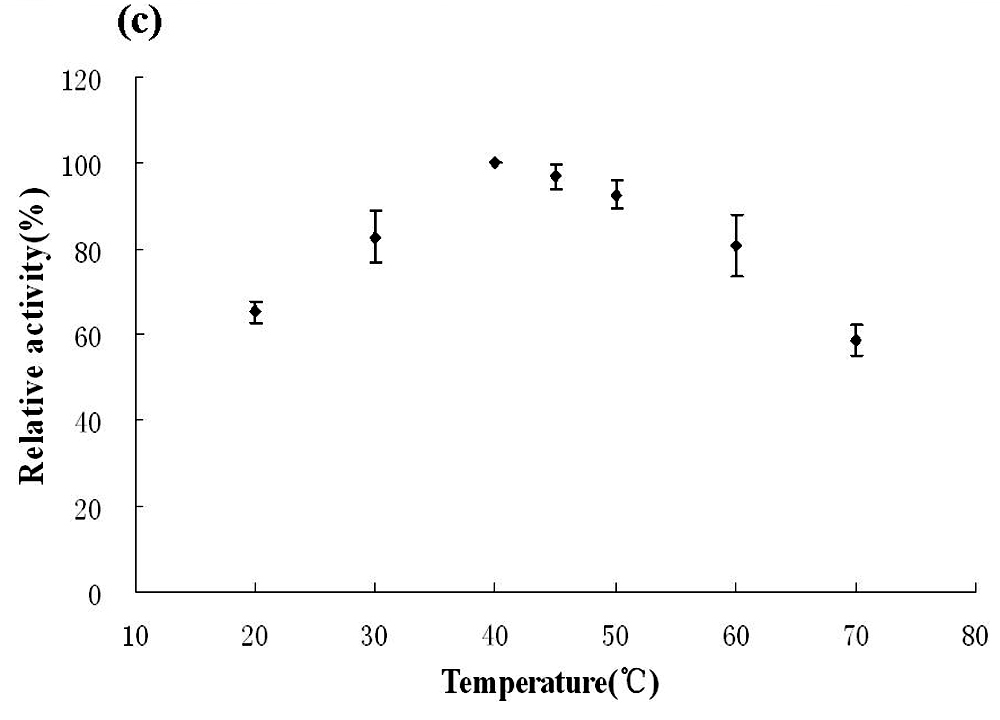
**

**
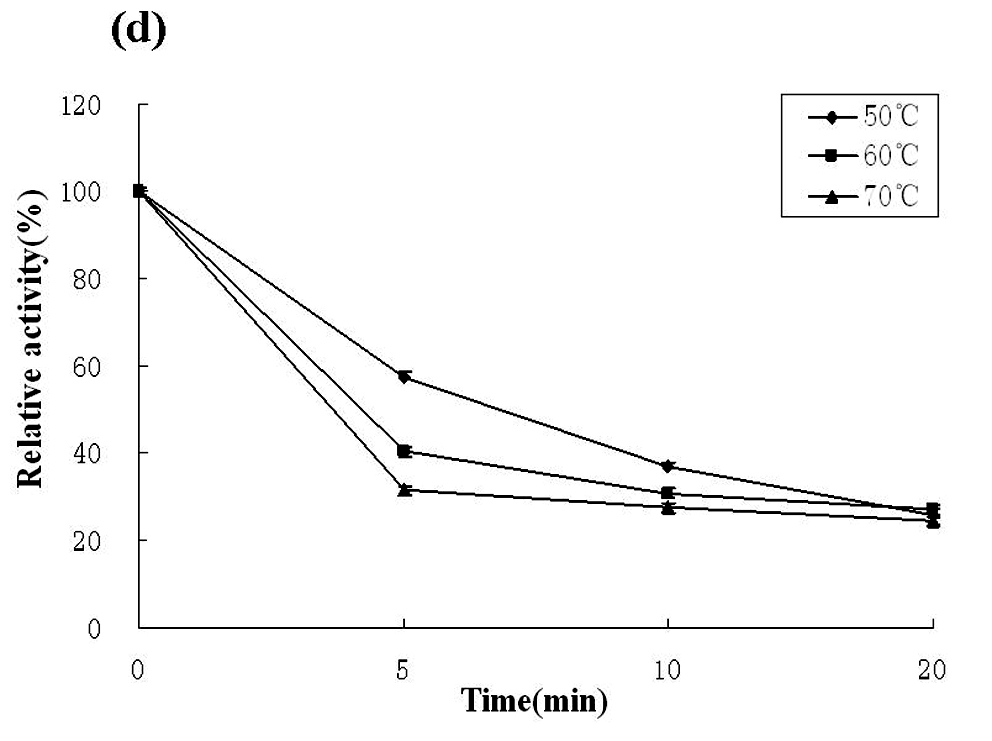
**

**Figure S3. The temperature and pH properties of His_6_-PdcDE.**

(a) Effects of pH on the activity of His_6_-PdcDE; (b) The pH stability of His_6_-PdcDE; (c) Effects of temperature on the activity of His_6_-PdcDE; (d) The temperature stability of His_6_-PdcDE.

**
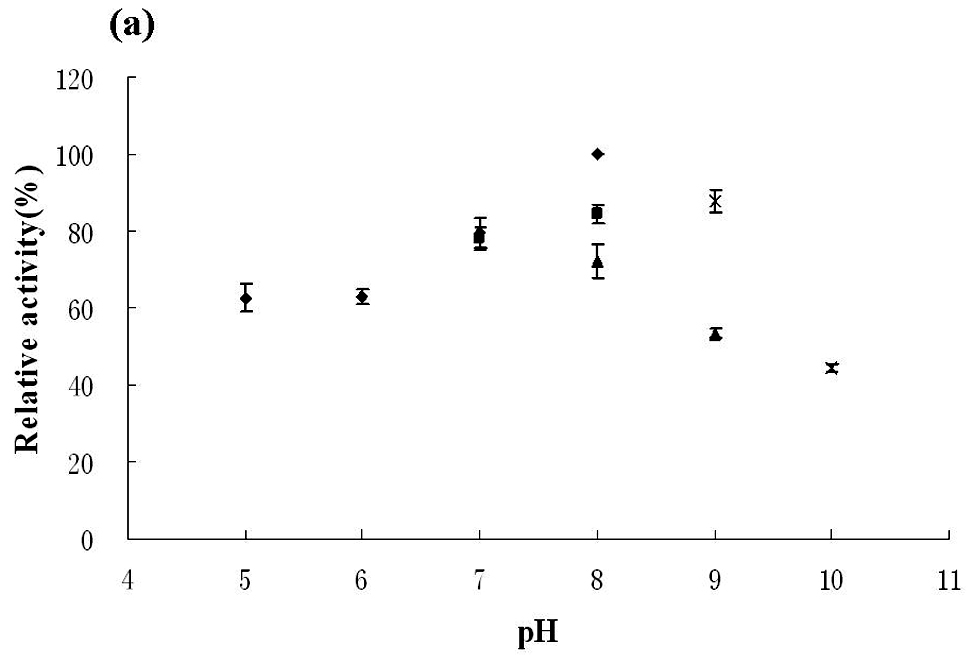
**

**
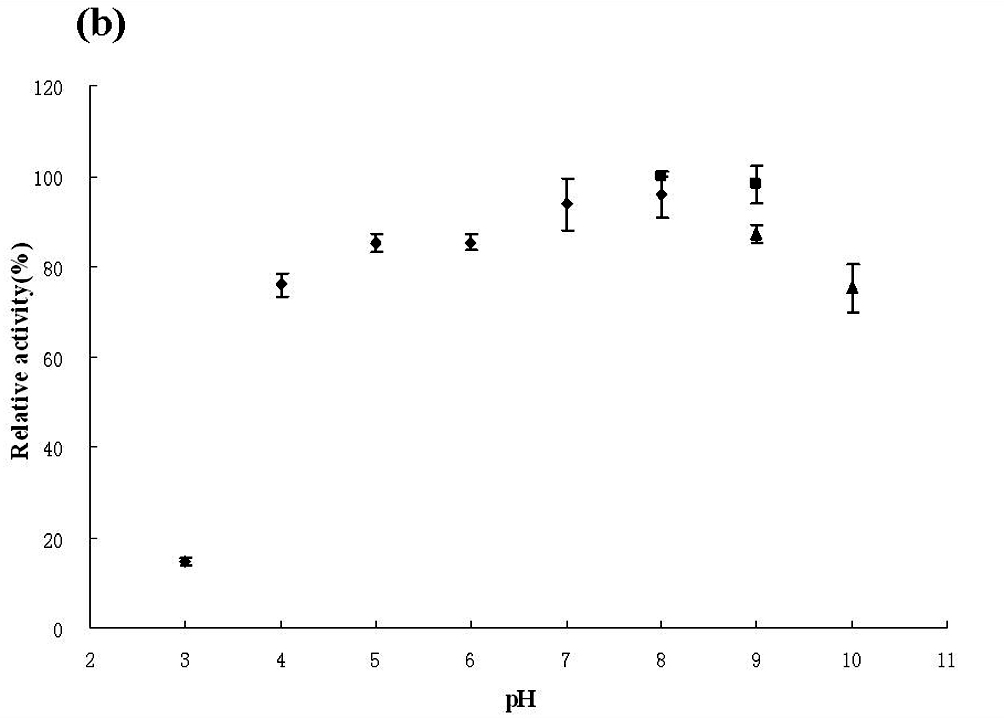
**

**
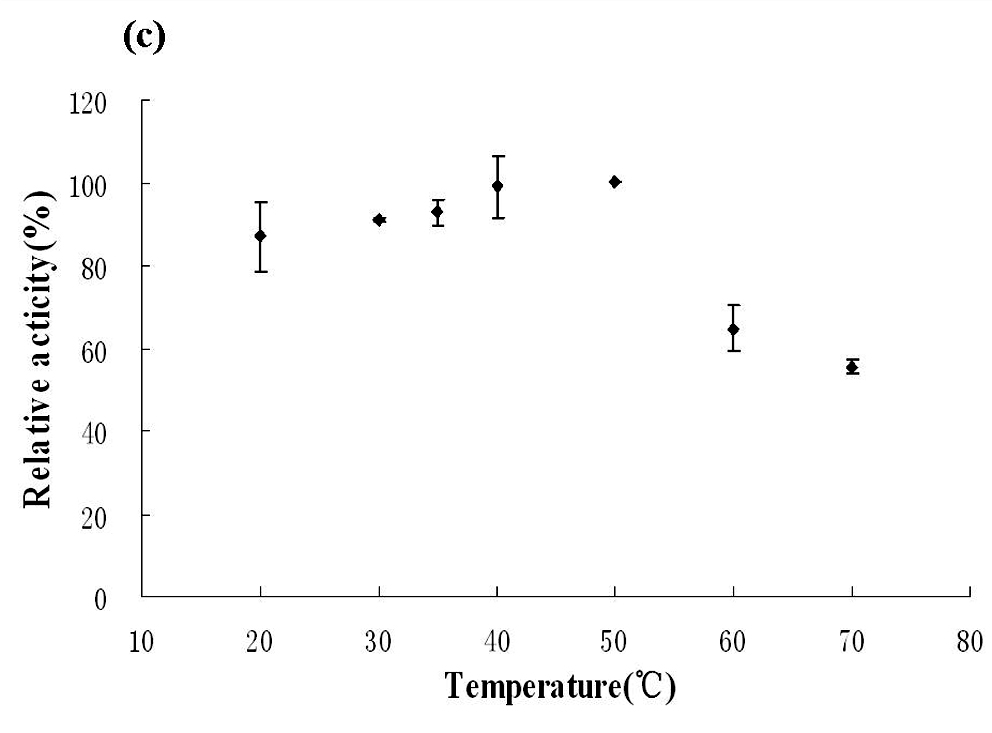
**

**
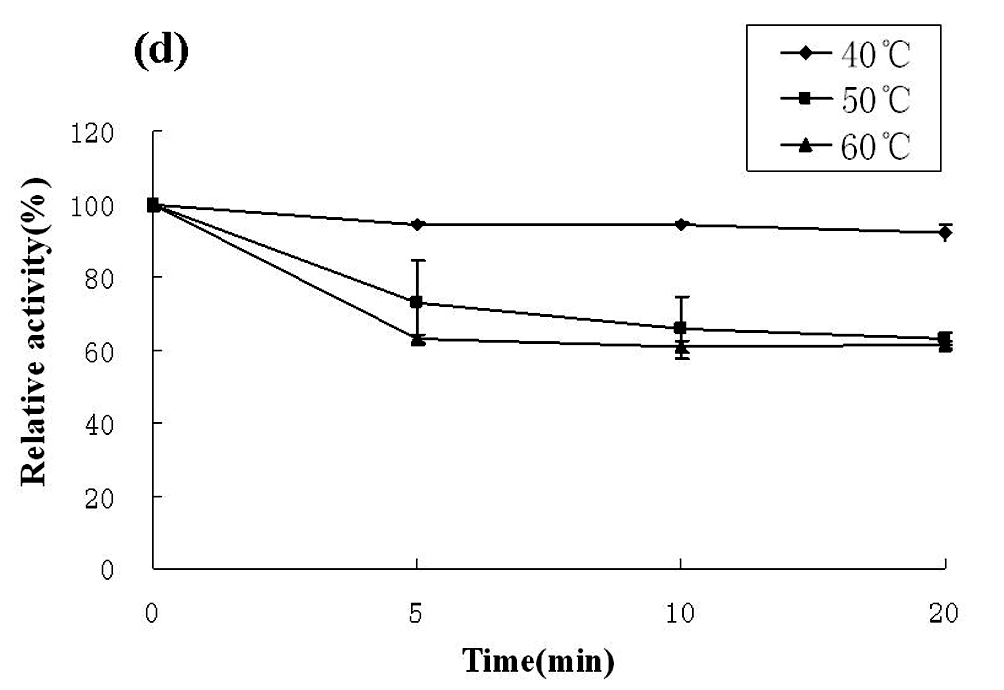
**

**Figure S4. The temperature and pH properties of His_6_-PdcG.**

(a) Effects of pH on the activity of His_6_-PdcG; (b) The pH stability of His_6_-PdcG; (c) Effects of temperature on the activity of His_6_-PdcG; (d) The temperature stability of His_6_-PdcG.

**
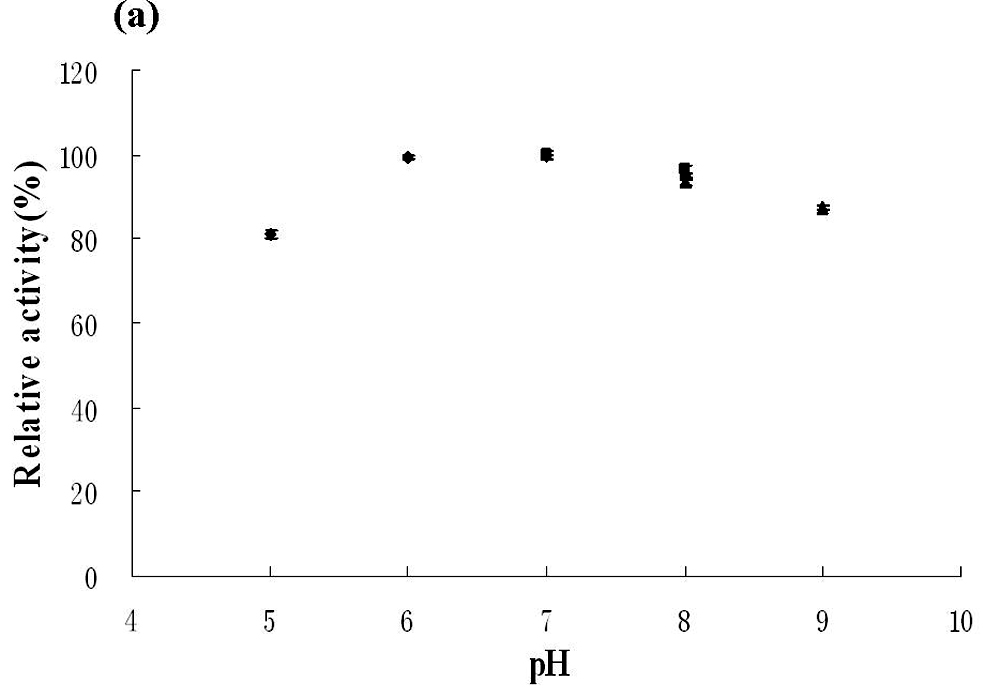
**

**
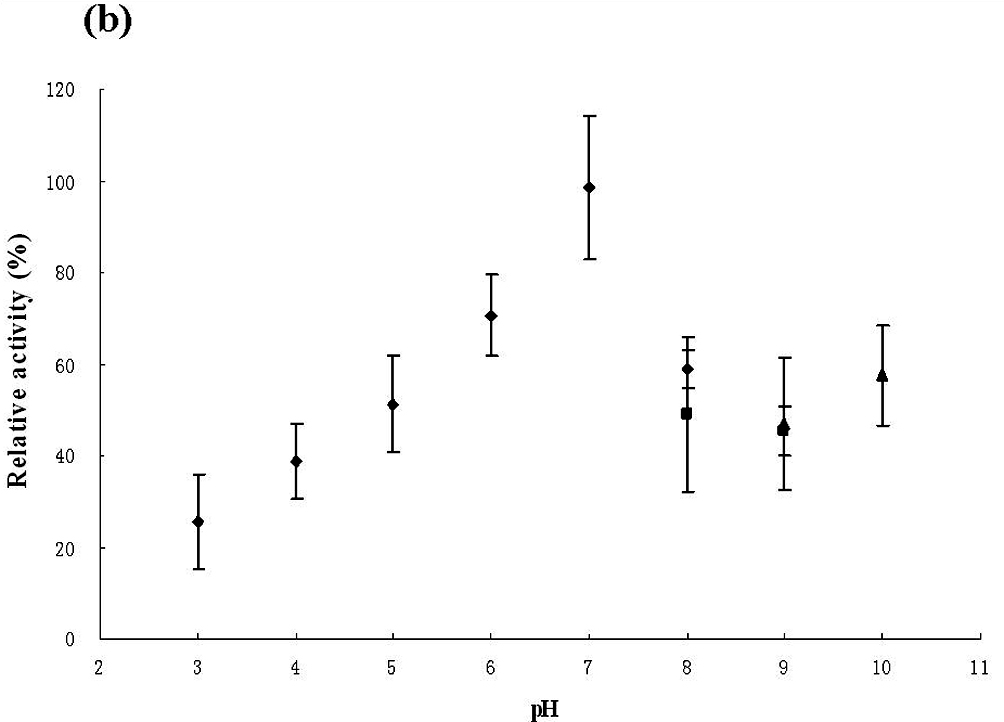
** **
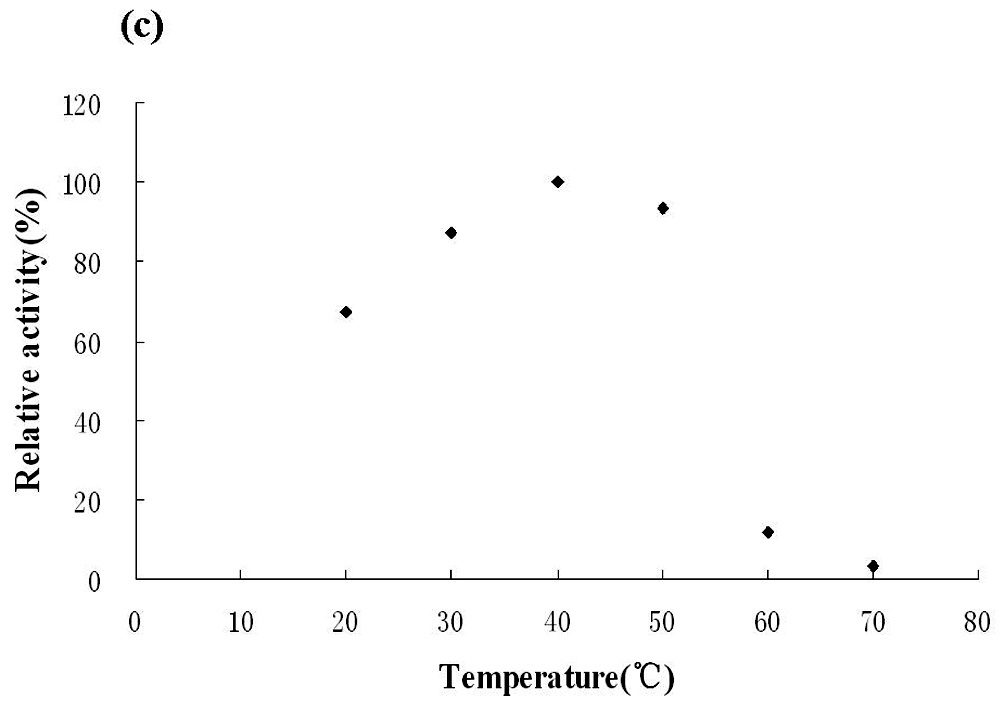
**

**
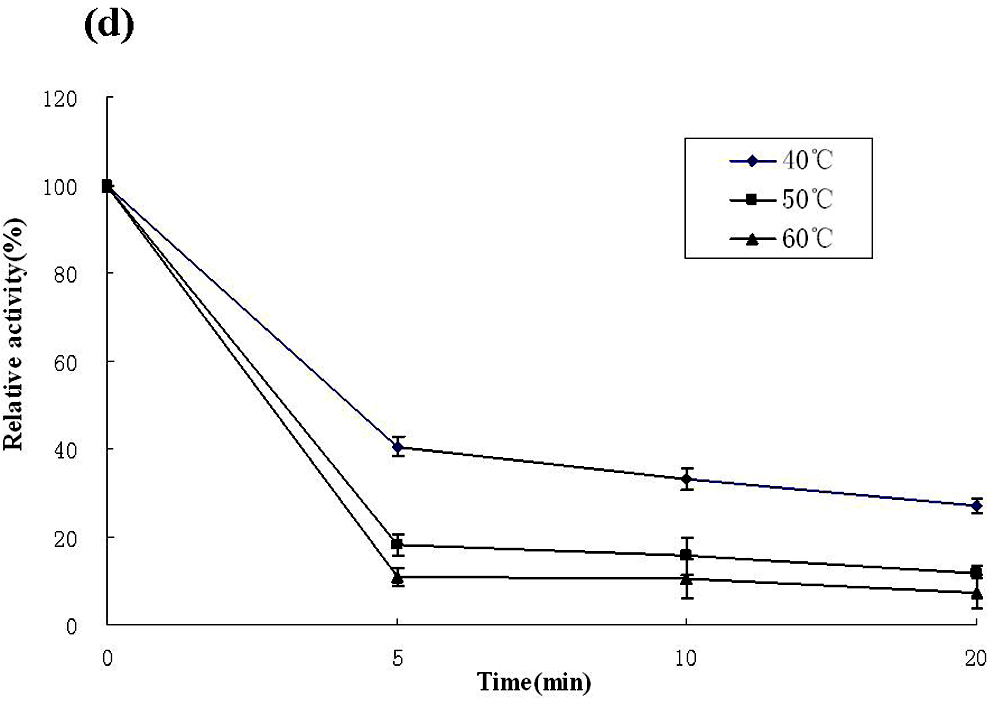
**

**Figure S5. The temperature and pH properties of His_6_-PdcF.**

(a) Effects of pH on the activity of His_6_-PdcF; (b) The pH stability of His_6_-PdcF; (c) Effects of temperature on the activity of His_6_-PdcF; (d) The temperature stability of His_6_-PdcF.
